# What should we be measuring when we measure cognitive reserve? Distinguishing proxies, prediction, and mechanism

**DOI:** 10.3389/fpsyg.2026.1972944

**Published:** 2026-09-18

**Authors:** Kenneth R. Paap

**Affiliations:** Department of Psychology, San Francisco State University, San Francisco, CA, United States

**Keywords:** cognitive aging, cognitive modeling, cognitive reserve, proxies, resillience

## Abstract

Cognitive reserve (CR) was proposed to explain why individuals with comparable brain aging or pathology can show different cognitive outcomes. Yet progress in CR assessment has been complicated by ambiguity about what, exactly, is being measured. This Conceptual Analysis distinguishes three elements that are often conflated: experiences proposed to build or maintain reserve, prediction of cognitive outcomes, and the cognitive or neural mechanisms that might produce resilience. The widespread Cognitive Reserve Index questionnaire (CRIq) offers a standardized measure of education, occupational activity, and cognitively stimulating leisure, but combining proposed determinants of reserve does not necessarily measure reserve itself. Similarly, associations between CR proxies and cognitive outcomes establish predictive validity but do not demonstrate that those proxies buffer the cognitive consequences of neural deterioration. Physiological measures potentially move assessment closer to mechanism, but a neural correlate is informative only when its functional meaning and predicted direction are independently constrained. Stronger tests of CR should distinguish and, where possible, independently measure prior cognitive ability, life-course experiences, brain status or pathology, candidate cognitive or neural mechanisms, and cognitive outcomes. On this view, CR may be better understood not as a quantity residing within an individual that can be captured by a single score, but as a theoretically specified pattern of relationships among independently measurable variables. Progress in CR assessment therefore requires movement from proxy to prediction, from prediction to resilience, and from resilience to mechanism.

## 1 Introduction: what kind of thing is cognitive reserve?

Before asking how cognitive reserve (CR) should be measured, it is useful to ask what kind of thing CR is supposed to be. Contemporary definitions characterize CR in terms of the ability to maintain cognitive performance better than expected given age-related brain change, injury, or disease. This formulation makes an important commitment: if CR refers to a genuine capacity that alters how effectively an individual copes with neural insult, then it must ultimately be instantiated in some aspect of brain structure or function. As Stern (2002) observed in an early formulation of the construct, differences in cognitive processing attributed to reserve must have a physiological basis because cognitive function is ultimately mediated by the brain.

This point is conceptually simple but has important implications for assessment. Education, occupational complexity, leisure activity, and related life experiences may contribute to the development or maintenance of CR, but they are not themselves the neural capacity presumed to produce resilience. Likewise, better cognitive performance is an outcome that CR is invoked to explain, not a direct measure of the mechanism responsible for that performance. The distinction is therefore among proposed determinants of reserve, reserve itself, and the cognitive consequences attributed to reserve.

Paap (2024) made a similar argument in proposing that cognitive performance should ultimately be understood in terms of the structural and functional capacities of the brain. On this view, experiences that enhance later cognition are theoretically interesting because they may modify brain structure or function, not because their occurrence can simply be relabeled as “reserve.” The problem becomes especially clear with conventional CR proxies. Education, occupation, and bilingualism are indirect indicators whose theoretical significance depends on the hypothesis that they produce changes in brain structure or function that subsequently support cognition in the face of aging or pathology.

Recent attempts to sharpen CR terminology have moved importantly in this direction. Stern et al. (2020), for example, distinguished cognitive reserve from brain reserve and brain maintenance and emphasized that life-course proxies should not be treated as direct measures of CR. This clarification represents an important conceptual advance. It also invites a further question: once proposed determinants of reserve are distinguished from reserve itself, what evidence would demonstrate that those experiences have actually produced or maintained a mechanism that increases cognitive resilience? Empirical studies nevertheless frequently continue to describe proxy scores as measures of the amount of CR an individual possesses. The distinction matters because increasingly sophisticated measurement of CR proxies does not necessarily provide increasingly direct measurement of CR. A proxy may predict cognition. A composite of several proxies may predict cognition better. A proxy may even correlate with a physiological variable. None of these findings, by itself, establishes that the proxy enhanced or maintained a cognitive or neural mechanism that buffers the consequences of brain aging or pathology.

At the time this Conceptual Analysis was invited, five articles had been accepted for this Research Topic. These studies were therefore not selected on the basis of their findings; rather, they constitute the set of contributions available when this concluding perspective was developed. They provide useful illustrations of the measurement problem precisely because they approach CR assessment from different directions. Some examine associations between CR proxies and behavioral outcomes; others disaggregate the components of conventional proxy indices; one follows cognitive outcome after a neurological insult; and another searches for an electrophysiological correlate of CR. Rather than reviewing these studies comprehensively or giving equal analytical weight, selected findings are used to ask a narrower question: what evidence moves CR research from measuring proxies, to predicting cognitive outcomes, to identifying a cognitive reserve mechanism?

## 2 When a proxy becomes the construct

The Cognitive Reserve Index questionnaire (CRIq; Nucci et al., 2012) provides a particularly revealing example of the measurement problem. The CRIq was developed to integrate three experiences commonly associated with cognitive reserve—education, occupational activity, and cognitively stimulating leisure activity—into a standardized index. Its title described it as “a new instrument for measuring cognitive reserve,” and subsequent research has increasingly adopted that interpretation. Its influence has been remarkable. More than a decade after its introduction, the annual rate at which the CRIq is cited continues to increase, and it has now accumulated more than 1,000 Google Scholar citations. Its prominence is also evident in the present Research Topic, where all five articles considered here use the CRIq.

There are good reasons for this success. Education alone is an impoverished description of an individual’s lifetime cognitive experiences, and a standardized instrument that incorporates occupational and leisure activities offers greater breadth and comparability across studies. The concern is therefore not with the usefulness of the CRIq as a measure of life-course experiences. Rather, it is with a subtle transition that can occur when a useful proxy becomes widely adopted: *the measure of experiences hypothesized to produce CR* gradually becomes treated as a measure of CR itself.

Ou et al. (2025) provide an unusually explicit example. They describe the CRIq as an instrument that *“measure[s] cognitive reserve that individuals have accumulated throughout their lifespan,”* and later contrast their comprehensive CRIq measure with earlier studies employing individual proxies such as education or occupation. Indeed, they argue that the CRIq more comprehensively assesses the *“amount of cognitive reserve accumulated”* because it incorporates education, occupational activity, and leisure. But adding several proposed determinants of reserve together does not necessarily move measurement closer to reserve itself. It may simply provide a more comprehensive measure of its hypothesized determinants.

This distinction becomes important when CR proxy scores are used to predict outcomes. Carbone et al. (2026), for example, report that current and retrospective CR proxies statistically mediate some associations between views of aging and quality of life, and appropriately describe the proxies as a possible “*behavioral pathway*” linking the two. Such associations may be interesting and potentially useful without establishing a CR mechanism. Education, social and intellectual engagement, and other activities could influence quality of life through many pathways—including social interaction, physical activity, affect, economic resources, or sense of purpose—without first enhancing a neural capacity that protects cognition from aging or pathology. Even strong incremental prediction or statistical mediation by a CR proxy therefore would not, by itself, establish that cognitive reserve produced the association.

Magnani et al. (2026) illustrate a different difficulty with treating a composite proxy score as the construct. When education, occupation, and leisure components are examined separately, they do not necessarily show equivalent relationships with cognitive and psychological outcomes. Given the exploratory nature of that study, its small sample, and the heterogeneity inherent in subjective cognitive decline, the particular pattern of associations should not be overinterpreted. The more general lesson is methodological: combining several experiences into a single index does not demonstrate that those experiences are interchangeable indicators of a common underlying capacity. Indeed, the value of examining the components separately is precisely that it permits this assumption to be tested rather than built into the measurement procedure.

The widespread success of the CRIq therefore presents something of a paradox. It has improved the standardized measurement of experiences thought to contribute to CR, while potentially making it easier to overlook the distinction between those experiences and the mechanism they are hypothesized to produce. This distinction can be represented as one hypothesized CR pathway:

Life-course experiences → changes in cognitive/neural mechanisms → altered impact of brain aging or pathology → cognitive resilience.

A CRIq score primarily measures the experiences on the left side of this sequence. Demonstrating that the score predicts variables on the right side is important, but it does not identify the intervening mechanism. The next challenge for CR assessment is therefore not simply to measure its proposed determinants more comprehensively, but to determine whether they produce cognitive or neural change that alters how cognition responds to brain aging or neural insult.

## 3 Predicting cognition is not demonstrating cognitive resilience

If CR proxies are not themselves measures of reserve, perhaps their validity can be established by showing that they predict cognitive performance. Associations between education, occupational complexity, cognitively stimulating activity, and later cognition are certainly relevant to CR theory. But prediction alone leaves unresolved the question that motivated the reserve construct in the first place: why do individuals with comparable brain aging or pathology show different cognitive outcomes? A variable associated with higher cognitive performance need not be a variable that reduces the cognitive consequences of neural deterioration.

Montemurro et al. (2026) provide a useful illustration. In a large sample of 1,001 healthy adults ranging from 18 to 99 years of age, they identified a general cognitive factor and found that higher CRIq scores were associated with better performance on many cognitive measures. These are potentially informative individual-differences associations. However, the study was cross-sectional: different individuals of different ages were compared at a single point in time. Consequently, the conclusion that “High CR reduces decline and slows its progression” cannot be established by this design. Cross-sectional age differences are not within-person cognitive decline, and an association between CRIq and cognition does not establish that the experiences represented in the CRIq caused either the level or trajectory of cognitive performance.

There is a deeper identification problem. Education, occupational attainment, and patterns of cognitively stimulating activity are themselves associated with cognitive ability. Consequently, a CRIq–cognition association cannot distinguish cognitive differences produced by those experiences from cognitive differences that preceded and partly influenced selection into them. Prospective studies in which cognitive ability was measured early in life illustrate the importance of this distinction. Kremen et al. (2019), for example, found that after general cognitive ability measured in young adulthood was taken into account, education, occupational complexity, and later cognitive activities together accounted for less than 1% of the variance in later-life cognitive functioning. Such findings do not imply that education or subsequent experience is causally inert. They do show why later-life correlations between CR proxies and cognition cannot, by themselves, identify an experience-dependent reserve mechanism.

A more distinctive test follows from the defining phenomenon CR was proposed to explain. If CR refers to resilience in the face of neural deterioration or injury, then the critical evidence is not simply a main effect of a CR proxy on cognition. Rather, a candidate mechanism of reserve should alter the cognitive consequences of increasing pathology: the relationship between neural insult and cognition should be weaker as a function of an independently measured mechanism hypothesized to produce resilience. Statistically, this prediction can appear as an interaction between the candidate mechanism and the severity of pathology.

Ou et al. provide an unusually informative example because their longitudinal study comes considerably closer to this design. They followed 371 patients after acute ischemic stroke, repeatedly assessed cognition with the MoCA, measured proposed CR with the CRIq, and assessed stroke severity with the NIH Stroke Scale (NIHSS). Their theoretical formulation is explicit: CR is “the brain’s ability to compensate for the pathological change in the brain,” and individuals with greater reserve should withstand greater pathological damage while maintaining better cognitive ability.

CRIq significantly predicted cognitive performance: individuals with higher proxy scores tended to have better cognition following stroke. Stroke severity also predicted poorer cognition. But the theoretically more informative CRIq × stroke-severity interaction was not significant (B = 0.01, 95% CI [−0.002, 0.03]). Thus, the study provides evidence that the CRIq predicts post-stroke cognitive performance, but not that higher CRIq scores attenuate the relationship between stroke severity and cognitive outcome.

This null interaction should not be regarded as decisive evidence against CR. Interaction effects are generally more difficult to detect than main effects, and measurement error in either component of an interaction can further attenuate the observed effect (Aguinis, 1995; McClelland and Judd, 1993). These concerns are relevant here. NIHSS is a clinical severity scale rather than a direct measure of neural damage, and the sample consisted predominantly of patients with mild strokes, restricting the range over which moderation could be detected. Stroke volume and location may be more relevant measures of pathology, and Ou et al. appropriately call for longitudinal neuroimaging studies to test CR moderation more directly. Nevertheless, the observed interaction estimate was close to zero (*B* = 0.01), with a relatively narrow 95% confidence interval [−0.002, 0.03]. Thus, although the result cannot establish the absence of a buffering effect, it provides little positive evidence that higher CRIq scores attenuated the relationship between stroke severity and cognitive outcome.

The importance of their study is therefore methodological rather than adjudicative. It illustrates the difference between two questions that are too easily conflated: (1) Does a CR proxy predict cognition? (2) Does reserve buffer the cognitive consequences of neural insult? The first question can establish predictive validity. The second is much closer to the theoretical purpose of CR. A particularly strong design would go further by measuring prior cognitive ability, subsequent life experiences, a cognitive or neural mechanism, brain pathology or change, and cognitive outcome independently. Such a design could ask whether experiences proposed to build reserve predict change in the candidate mechanism beyond preexisting ability and, critically, whether that mechanism alters the relationship between measured brain change and cognitive change. That is a considerably more demanding standard than demonstrating a correlation between a CR proxy and cognition, but it is also the kind of evidence needed if CR is to function as a mechanistic rather than merely descriptive construct.

## 4 A neural correlate is not yet a neural mechanism

If cognitive reserve is ultimately instantiated in brain structure or function, then identifying a physiological marker of reserve would represent an important advance over reliance on life-experience proxies. Ušacka et al. (2026) move explicitly in this direction by asking whether the P300 event-related potential might provide a physiological signature of CR. Their study assessed CR with the CRIq, working memory with the Corsi block-tapping task, and P300 amplitude and latency during an auditory oddball task in healthy older adults and individuals with mild or moderate cognitive impairment. This is precisely the kind of attempt to move from presumed determinants of reserve toward a candidate neural manifestation that the field needs.

The difficulty is that a neural measure is informative about mechanism only if its functional meaning is sufficiently constrained. P300 amplitude illustrates the problem. A larger amplitude can be interpreted as greater recruitment of attentional or cognitive resources. Yet, under a neural-efficiency account, a smaller amplitude can potentially be interpreted as evidence that fewer neural resources are required to achieve comparable performance. Without an independently validated directional prediction, either result can be accommodated by a plausible post-hoc account. This is a form of valence ambiguity: neural differences are observed, but the direction that should count as greater efficiency or reserve is not uniquely specified in advance. More generally, valence ambiguity is a construct-validity problem. In the terminology of Cronbach and Meehl (1955), evidence for a construct derives meaning from theoretically specified relationships within a nomological network. If either an increase or a decrease in a candidate physiological marker can be interpreted after the fact as evidence for greater reserve, the observation provides little discriminating evidence for the construct.

The problem is not unique to electrophysiology. Functional neuroimaging can present a similar ambiguity when greater activation is interpreted as compensatory recruitment whereas reduced activation is interpreted as greater neural efficiency, unless theory or independent evidence specifies which pattern should be expected.

A related problem has been noted in other ERP literatures, where differences in amplitude or latency become theoretically meaningful only when they can be tied independently to task demands or behavioral performance (Paap et al., 2014; Cespón and Carreiras, 2020).

The findings of Ušacka et al. illustrate why this matters. In healthy controls, higher CR predicted larger P300 amplitude. In the MCI group, however, the CR coefficient at average working-memory capacity was negative, whereas the CR × working-memory interaction was positive. The association between CR and P300 amplitude also differed across cognitive-status groups. Estimates in the moderate-impairment group were unstable, and no reliable CR associations with P300 latency were observed in the healthy or MCI groups. These results may reflect genuine variation in neural processing across stages of cognitive impairment, but they do not identify a single physiological signature of reserve.

Unexpected interactions need not be uninformative. They can reveal important boundary conditions when the underlying measure has a well-established functional interpretation. Here, however, the meaning of P300 amplitude is already theoretically ambiguous. Adding interactions with working memory and cognitive status increases the number of possible interpretations without resolving which neural process constitutes reserve. A correlation between CRIq and P300 therefore establishes that a composite of life experiences covaries with an electrophysiological measure; it does not establish that P300 amplitude indexes neural efficiency or that neural efficiency is the mechanism through which those experiences produce cognitive resilience.

This distinction is especially important because the move from behavioral proxies to EEG can create an impression of greater mechanistic precision. Better technology, however, does not necessarily produce better construct validity. A neural marker of CR should ideally satisfy at least three conditions: its functional interpretation should be supported by evidence independent of the CR association being tested, such as experimental manipulation, converging behavioral evidence, or prior validation; the direction of the predicted effect should follow *a priori* from theory or independent evidence rather than from the observed result; and variation in the marker should help explain why individuals with comparable brain pathology differ in cognitive outcome. Ušacka et al. should therefore be viewed less as evidence that P300 is a physiological measure of CR than as an instructive demonstration of what remains to be established. Their study takes the important step of searching for a neural manifestation rather than relying exclusively on distal proxies.

Taken together, the preceding sections suggest a simple progression. If cognitive reserve is a genuine construct, it must ultimately be instantiated in brain structure or function. Measures such as the CRIq assess experiences hypothesized to influence reserve, not reserve itself. Associations between those proxies and cognition establish prediction, but not necessarily resilience to neural insult. And neural correlates become mechanistically informative only when their functional significance is independently constrained. These distinctions point toward a more demanding question: what would count as a convincing assessment of cognitive reserve?

## 5 What would count as a convincing assessment of cognitive reserve?

The distinctions developed above suggest that progress in CR assessment requires more than increasingly comprehensive proxy measures or increasingly sophisticated correlates of those measures. If CR is proposed as a mechanism through which experience produces resilience to brain aging or pathology, then the components of that claim should, wherever possible, be measured separately. At minimum, this requires distinguishing prior cognitive ability, experiences proposed to build or maintain reserve, brain status or pathology, candidate mechanisms of reserve, and cognitive outcomes. No single study need measure all five components, but clarity about which component is being measured—and which causal link is being tested—would substantially strengthen inference.

Prior cognitive ability is particularly important because it precedes many of the experiences subsequently treated as determinants of reserve. Individuals do not enter educational, occupational, and leisure environments randomly. Cognitive ability influences educational attainment, occupational selection and success, and likely the kinds of cognitively stimulating activities people pursue. These relationships need not be unidirectional: subsequent educational, occupational, and leisure experiences may in turn influence cognitive development or maintenance, and cognitive performance may influence continued engagement in such activities. Longitudinal associations between experience and cognition can therefore reflect selection, experience-dependent change, and reciprocal influence. Genetic influences add another source of nonrandom variation: shared genetic influences on cognitive ability, educational attainment, and related life-course outcomes can contribute to associations between CR proxies and later cognition.

As the prospective evidence reviewed above illustrates, measuring prior cognitive ability directly can substantially reduce associations otherwise attributed to later reserve-building experiences. This does not imply that experience is unimportant. It shows that associations between life-course experiences and later cognition can substantially reflect individual differences that preceded those experiences. A reserve-building hypothesis therefore requires evidence that experience predicts subsequent change beyond what would be expected from prior cognitive ability and correlated developmental circumstances.

Experiences proposed to influence reserve should likewise be treated as exposures rather than as reserve itself. Education, occupation, social and intellectual engagement, physical activity, and multilingual experience may have different effects, operate during different developmental periods, and act through different pathways. Combining them into a composite can be useful for prediction but risks assuming the very common mechanism that CR theory needs to establish. Whenever possible, these experiences should therefore be measured separately and their effects compared rather than presuming that each contributes interchangeable units to a common reserve.

Life-course experiences may also influence later cognitive outcomes through brain maintenance rather than, or in addition to, cognitive reserve. In the Reserve and Resilience Framework (Stern et al., 2023), brain maintenance refers to reduced development of age-related brain changes or pathology, whereas CR concerns individual differences in cognitive performance associated with a given level of such brain change. Thus, an experience associated with both healthier brain status and better cognition need not have produced a mechanism that buffers the cognitive impact of pathology; it may instead have reduced the pathology to be buffered. Distinguishing these pathways requires measurement of brain change as well as cognitive outcome.

Brain status or pathology provides the reference against which resilience can be evaluated. The central CR phenomenon is not simply good cognition but cognition that is better than expected given the degree of neural aging, injury, or disease. Consequently, direct measures of relevant brain change—such as lesion characteristics, structural change, disease biomarkers, or appropriately validated functional measures—are preferable to assuming resilience from cognitive performance alone. Longitudinal measurement is especially valuable because it permits changes in brain status and cognition to be related within individuals rather than inferred from cross-sectional age differences.

Candidate cognitive or neural mechanisms occupy the critical middle of the causal chain. If an experience builds or maintains reserve, something must change that enables cognition to withstand neural deterioration more successfully. Candidate mechanisms might involve efficiency, flexibility, network organization, compensatory recruitment, or other processes, but naming such a process is not sufficient. The measure chosen to represent it should have independently established functional meaning, and the direction of the predicted effect should ideally be specified before the data are observed. Otherwise, the same neural difference can too readily be interpreted as either greater efficiency or greater compensatory recruitment depending on the observed result.

Finally, cognitive outcomes should be distinguished from reserve itself. Higher cognitive performance may be consistent with greater reserve, but it can arise for many other reasons. The stronger test asks whether cognition is better maintained than would be expected from prior ability and measured neural change. In this sense, the crucial outcome is not simply cognitive level but resilience conditional on neural insult.

These components suggest a hierarchy of increasingly informative evidence. An association between a CR proxy and cognition demonstrates prediction. Evidence that the relationship between brain pathology and cognition differs as a function of an independently measured candidate mechanism provides a more direct test of resilience or buffering. Stronger still would be evidence that a life-course experience predicts change in an independently validated cognitive or neural mechanism, which in turn accounts for reduced cognitive consequences of subsequent brain change. Such a design begins to test the theoretical sequence illustrated in Figure 1.

**Figure 1:**
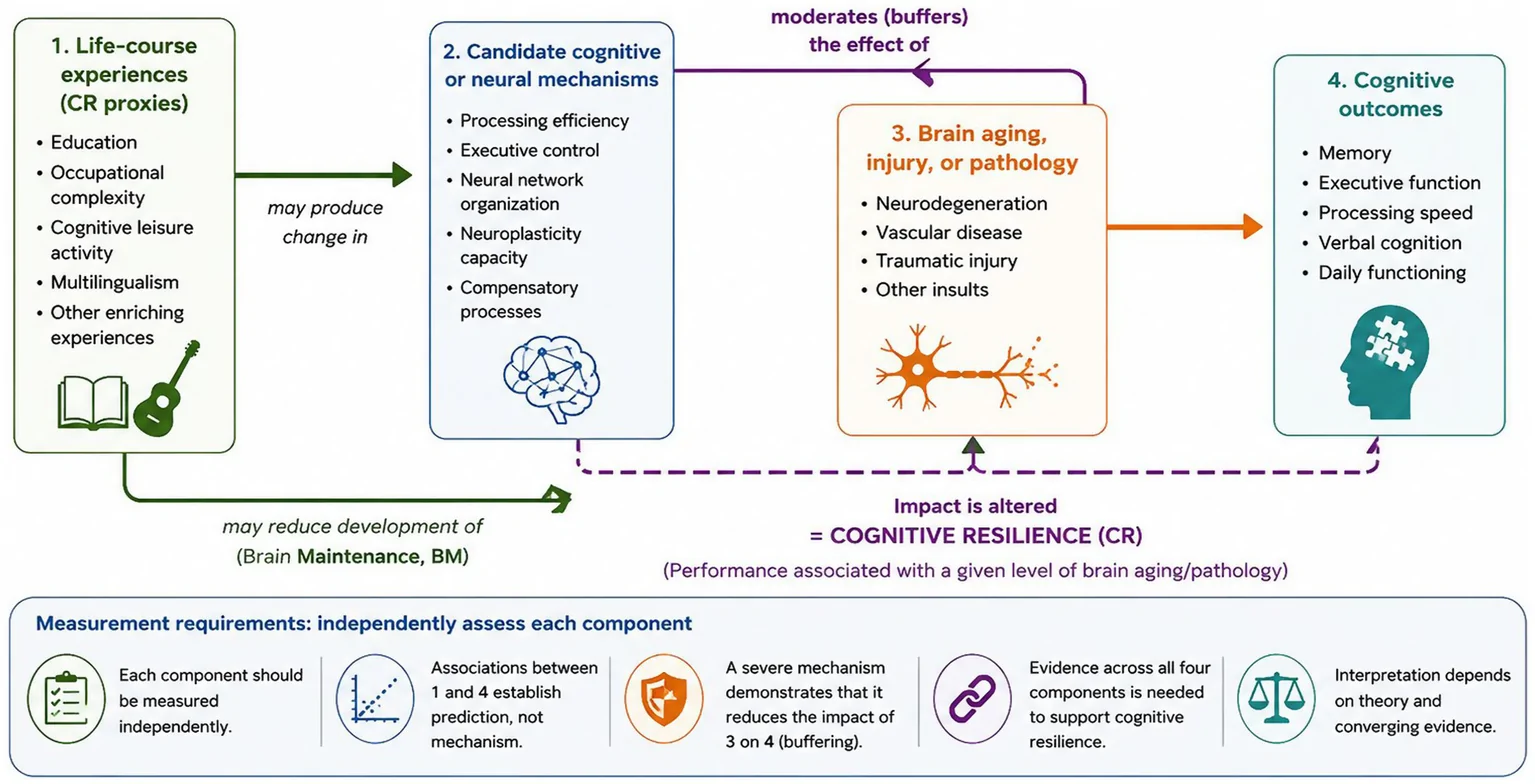
From cognitive-reserve proxies to a testable reserve mechanism. A theoretical framework with dual pathways (reserve and maintenance) and key measurement requirements. Life-course experiences (1) can influence cognitive outcomes (4) through two pathways. One pathway (purple) is cognitive reserve (CR): experiences may build or maintain mechanisms (2) that moderate the effect of brain aging, injury, or pathology (3) on cognition. The other pathway (green) is brain maintenance (BM): experiences may reduce the development or progression of brain aging or pathology. Selection effects and reciprocal relationships (e.g., prior ability influencing experiences; performance influencing subsequent activity) are not depicted for clarity.

This framework should not be interpreted as requiring every CR study to estimate this entire sequence. Different designs can provide evidence about different links. A longitudinal cohort with childhood cognitive testing may be especially informative about selection and antecedent ability; a stroke study may provide an unusually strong test of buffering; neuroimaging or electrophysiology may help identify candidate mechanisms. The important requirement is conceptual rather than technological: the variable being measured should not be asked to serve simultaneously as the presumed cause of reserve, the amount of reserve possessed, and evidence that reserve has produced its expected consequence.

These components also imply distinct measurement requirements. Prior cognitive ability should ideally be measured prospectively or estimated independently of the later cognitive outcome. Life-course experiences should be measured as experiences rather than assumed to constitute reserve. Brain status or pathology should be assessed independently, using measures sufficiently sensitive to the neural change relevant to the population under study and with enough variation to test whether its cognitive consequences differ across individuals. Candidate cognitive or neural mechanisms require measures whose functional interpretation and predicted direction are supported independently of the CR association being tested. Finally, cognitive outcomes should be measured separately from both the proposed mechanism and the experiences hypothesized to influence it. The particular instruments will necessarily vary with the population, pathology, and hypothesized mechanism; the critical requirement is that the components not be operationally collapsed into one another.

## 6 Discussion: from measuring reserve to testing reserve

The studies considered here illustrate both the progress and the continuing difficulty of cognitive reserve assessment. Standardized instruments such as the CRIq have improved measurement of life experiences proposed to contribute to reserve. Longitudinal studies have begun to examine cognition following neurological insult, and physiological approaches seek neural signatures that might move CR research closer to mechanism. These are important developments. Yet they also expose a common inferential problem: proposed determinants of reserve, predictors of cognitive outcomes, and mechanisms of resilience are not interchangeable measures of the same construct.

A different conception may be more productive. Cognitive reserve need not be treated as a thing residing within an individual that can be captured by a single score. It may be better understood as a theoretically specified pattern of relationships among things that can be measured independently: prior cognitive ability, life-course experiences, brain structure and function, pathology or age-related brain change, candidate cognitive and neural mechanisms, and cognitive outcomes. This does not negate the requirement that any process producing cognitive resilience must ultimately have a neural implementation; rather, it distinguishes those measurable processes from CR as a separate quantity presumed to reside in the brain. On this view, the empirical signature of reserve is not high education, a high CRIq score, superior cognitive performance, or a particular neural signal. It is evidence that cognition is better maintained than expected given measured brain change, together with evidence identifying the processes through which that resilience occurs.

Several priorities follow for future research. Prospective studies should measure antecedent cognitive ability before, or independently of, the life-course experiences hypothesized to build reserve, thereby improving separation of selection from experience-related change. Longitudinal designs should measure both brain change and cognition so that brain maintenance—reduced development of pathology—can be distinguished from cognitive reserve—better cognitive performance at a given level of pathology. Tests of resilience should be designed with sufficient variation in brain status and sufficient statistical power to detect moderation rather than relying primarily on main effects of CR proxies. Finally, candidate cognitive or neural mechanisms should generate directional predictions that are independently constrained and tested against measured brain change and cognitive outcome. Converging evidence across these components would provide a substantially stronger basis for inferring a reserve mechanism than any proxy score, cognitive association, or neural correlate considered alone.

This perspective also changes what should count as progress in CR assessment. A better questionnaire may measure proposed exposures more reliably. A better cognitive battery may measure outcomes more precisely. Neuroimaging and electrophysiology may provide increasingly sensitive measures of brain function. But none, considered alone, necessarily provides a better measure of reserve. The stronger advance comes from designs that separate these variables and test the relationships among them—including alternative explanations based on prior cognitive ability, developmental selection, socioeconomic circumstances, and health.

The first five studies in this Research Topic approach different parts of this problem and, collectively, make the remaining challenge especially visible. Advancing cognitive reserve assessment requires movement from proxy to prediction, from prediction to resilience, and from resilience to mechanism. The goal should therefore be less to identify the measure of cognitive reserve than to specify and test the pattern of evidence that would demonstrate that reserve is doing explanatory work.
